# Bioinformatics analysis identifies immune-related gene signatures and subtypes in diabetic nephropathy

**DOI:** 10.3389/fendo.2022.1048139

**Published:** 2022-12-07

**Authors:** Kunna Lu, Li Wang, Yan Fu, Guanghong Li, Xinhuan Zhang, Mingfeng Cao

**Affiliations:** ^1^ Department of Endocrinology, The Second Affiliated Hospital of Shandong First Medical University, Taian, Shandong, China; ^2^ Department of Pharmacy, The Second Affiliated Hospital of Shandong First Medical University, Taian, Shandong, China; ^3^ The Second Affiliated Hospital of Shandong First Medical University, Taian, Shandong, China

**Keywords:** diabetic kidney disease, bioinformatics, immune infiltration, xCell, GSEA

## Abstract

**Background:**

Systemic inflammation and immune response are involved in the pathogenesis of diabetic nephropathy (DN). However, the specific immune-associated signature during DN development is unclear. Our study aimed to reveal the roles of immune-related genes during DN progression.

**Methods:**

The GSE30529 and GSE30528 datasets were acquired from the Gene Expression Omnibus (GEO) database. Then, the intersection between differentially expressed genes (DEGs) and immune score-related genes (ISRGs) was screened. Subsequently, functional enrichment analyses were performed. The different immune phenotype-related subgroups were finally divided using unsupervised clustering. The core genes were identified by WGCNA and the protein-protein interaction (PPI) network. xCell algorithm was applied to assess the proportion of immune cell infiltration.

**Results:**

92 immune score-related DEGs (ISRDEGs) were identified, and these genes were enriched in inflammation- and immune-associated pathways. Furthermore, two distinct immune-associated subgroups (C1 and C2) were identified, and the C1 subgroup exhibited activated immune pathways and a higher percentage of immune cells compared to the C2 subgroup. Two core genes (LCK and HCK) were identified and all up-regulated in DN, and the expressions were verified using GSE30122, GSE142025, and GSE104954 datasets. GSEA indicated the core genes were mainly enriched in immune-related pathways. Correlation analysis indicated LCK and HCK expressions were positively correlated with aDC, CD4+ Tem, CD8+T cells, CD8+ Tem, and mast cells.

**Conclusions:**

We identified two immune-related genes and two immune-associated subgroups, which might help to design more precise tailored immunotherapy for DN patients.

## Introduction

Diabetes accounts for 30% to 50% of all chronic kidney disease cases, affecting 285 million people worldwide (1). Diabetic nephropathy (DN) induced by diabetes mellitus is the leading cause of end-stage kidney disease in both developing and developed countries (2). Microalbuminuria is one of the important indicators to evaluate the development of DN in clinical practice (3). Studies now indicated that not all diabetic patients with renal failure have massive proteinuria (4). It is not accurate to assess the prognosis or severity simply based on the degree of proteinuria (5). Besides, the current treatment strategies for DN are aimed at controlling blood pressure and blood glucose levels and suppressing the RAS system to slow DN development (6, 7). However, due to the individual heterogeneity of DN, not all patients could obtain effective treatment effects. Therefore, it is critical need to identify novel biomarkers and develop new methods for the early diagnosis and treatment of DN patients.

Given the morbidity and mortality associated with DN, many studies have sought to investigate the pathogenesis of DN and promote drug development aimed at showing or revering the DN progression. Previous studies revealed that renal fibrosis, activation of the renin-angiotensin-aldosterone system, oxidative stress, and inflammation are the major pathogenesis features of DN (8–10). Also, mitochondria dysfunction, autophagy, and innate immunity are implicated in the development of DN (11–13). The multiple signaling pathways are involved in the immune pathogenesis of DN (14). In diabetes, high blood sugar and high levels of lipids (including oxidized lipids, reactive oxygen species, and oxidative stress), cause the generation of damage-related molecular patterns, and then activate the inflammation-related pathways (15). In response to chronic activation of immune damage, podocytes, endothelial cells, and renal mesangial cells could generate all kinds of inflammatory factors, such as adhesion molecules, chemokines, and cytokines, which recruit macrophages and monocytes, and further initiate the pro-inflammatory cascades (16). A recent study showed that tissue-infiltrated immune cell populations play an important role in the pathogenesis of DN, including the specific contributions of leucocyte subsets (mast cells, lymphocytes, neutrophils, and macrophages) (17–19). Besides, the immune infiltration pattern was significantly changed in the glomerulus of DN (20). Furthermore, IDO1 was identified as a novel immune-related marker for DN patients and revealed to be involved in immune cell infiltrates in DN (21). Therefore, investigating the immune mechanisms of DN could provide novel insights into DN pathogenesis.

**Figure 1:**
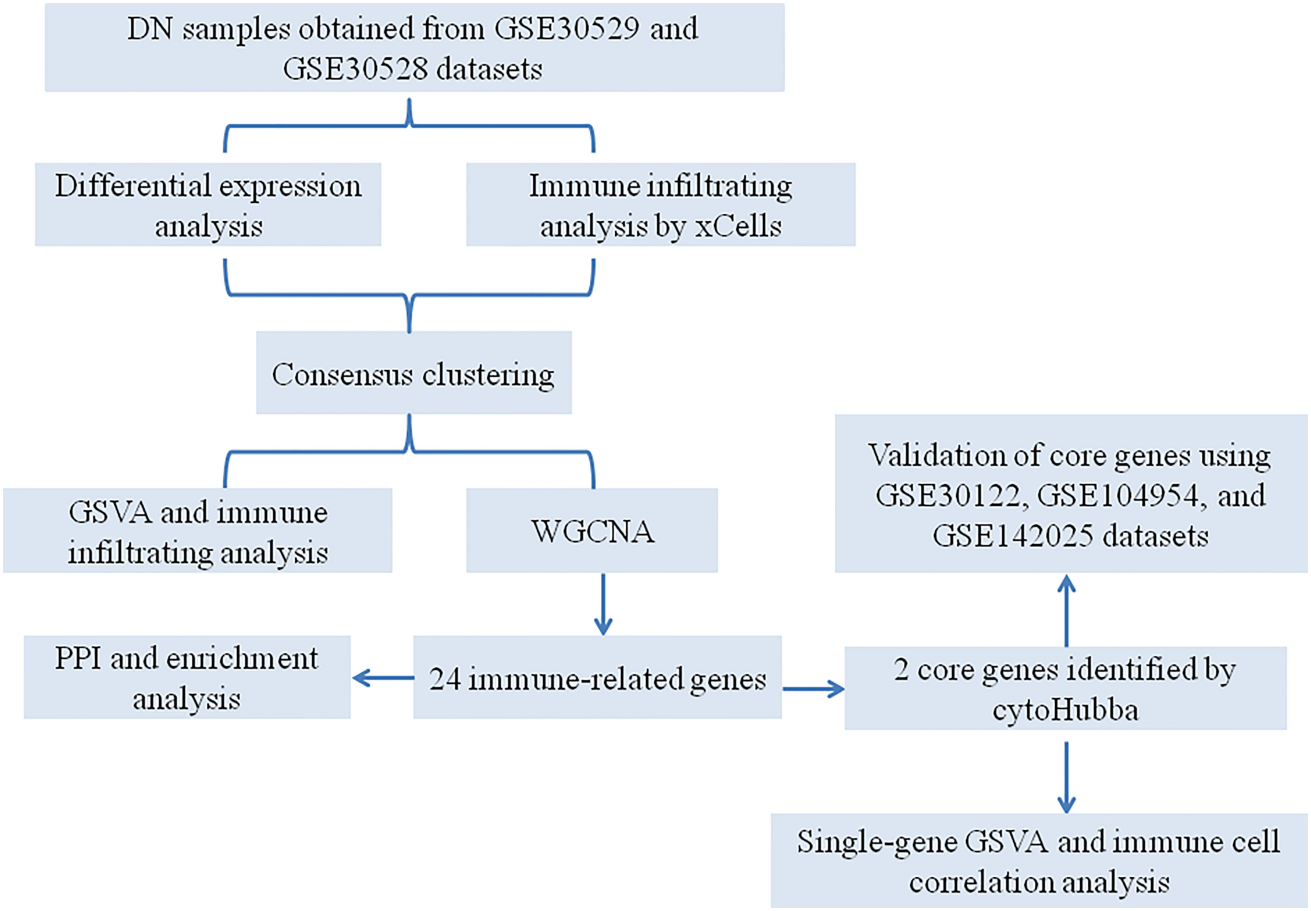
A flowchart for the analysis process to identify potential genes and molecular subgroups in this study.

With the rapid development of genomics and bioinformatics, many disease databases have been established and improved, which provided a theoretical basis for exploring the new therapeutic targets and pathogenesis of diseases. Identification of immune cell infiltration-related differentially expressed genes *via* bioinformatics analysis could provide potential biomarkers for the diagnosis of DN and help us to better understand the pathogenesis of DN (22, 23). In this research, we aimed to identify immune-related genes (IRGs) that are implicated in the DN progression *via* a series of bioinformatics methods. Furthermore, unsupervised clustering was used to identify immune phenotype-associated subtypes in DN patients. Our findings will provide the theoretical basis for a better understanding of the immune response associated with DN development. The workflow of this study is presented in **Figure 1**.

## Materials and methods

### Collection of datasets

A total of five gene expression profile datasets, namely GSE30529, GSE30528, GSE104954, GSE142025, and GSE30122, were acquired from the Gene Expression Omnibus (GEO, available at: https://www.ncbi.nlm.nih.gov/geo/). The details for these datasets are presented in **Table 1**. Before data analysis, in order to remove possible outliers and make sure the accuracy of data, all gene expression profiles were standardized and normalized using limma R package (24). Two microarray datasets (GSE30529 and GSE30528) were merged and used as the training dataset. The surrogate variable analysis (SVA) algorithm was used to eliminate the batch effects (25).

**Table 1:** The microarray dataset information.

Dataset ID	Platform	DN		Normal		Data type
		Type	Number	Type	Number	
GSE30528	GPL571	Glomeruli	9	Glomeruli	13	Training
GSE30529	GPL571	Tubuli	10	Tubuli	12	Training
GSE104954	GPL22945	Tubulointerstitium	7	Tubulointerstitium	18	Testing
GSE104954	GPL24120	Tubulointerstitium	10	Tubulointerstitium	3	Testing
GSE142025	GPL20301	Kidney biopsies	21	Kidney biopsies	9	Testing
GSE30122	GPL571	Glomerulus	19	Glomerulus	50	Testing

### A screen of immune score-related DEGs

First, we used the limma package of R to screen DEGs between the normal and DN groups (24). ∣logFC∣≥ 1 and p.adj < 0.05 were the cutoff criteria for DEGs screen (26). The immune score of each sample was calculated by xCell algorithm (27). Besides, the training dataset of samples was divided into the high immune score and low immune score groups based on the median values of the immune score. Immune score-related genes (ISRGs) were screened between the two immune score-associated groups using the limma package of R. ∣logFC∣≥ 1 and p.adj < 0.05 were the cutoff criteria for ISRGs identification. The ggplot and heatmap packages of R were used to visualize the analysis results. ISRDEGs were obtained by the intersection of DEGs and ISRGs.

### Unsupervised clustering analysis

Based on the gene expression profiles of ISRDEGs, we further performed a consensus cluster analysis to identify distinct immune cell infiltration-related subgroups. In this study, the robustness and clustering number were evaluated by using a consensus clustering algorithm (28). The “ConsensusClusterPlus” package of R was applied to perform 1000 iterations to ensure the robustness of the classification. The maximum cumulative distribution function (CDF) index was used as the optimal K value.

### Weighted gene coexpression network analysis

WGCNA was used to establish potential modules associated with different subgroups of DN samples (29). The distance between each gene was calculated by the Pearson correlation coefficient, WGCNA package was applied to establish the correlation adjacency matrix. The hierarchical clustering analysis was performed using the “hclust” function, and the “pickSoftThreshold” function was applied to calculate the soft thresholding power value. Furthermore, the associated modules were constructed using the “blockwiseModules” function. The hub genes in the most relevant modules were identified based on Module Membership (MM) > 0.8 and Gene Significance (GS) > 0.6 (30).

### Functional enrichment analysis and protein-protein interaction

A total of 1793 immune-related genes (IRGs) were collected from the InnateDB website (https://www.innatedb.com/redirect.do?go=resourcesGeneLists). The clusterProfiler package of R was applied to perform the Kyoto Encyclopedia of Genes and Genomes (KEGG) pathway and Gene Ontology (GO) annotation enrichment analyses (31). For the top 10 significant GO and KEGG pathways, the ggplot2 R package of R was used to draw the bubble diagram. The Search Tool for the Retrieval of Interacting Genes (STRING) database (https://string-db.org/) was used to construct the PPI network, and the medium confidence > 0.4 was set to generate the TSV format file (32). Then, the PPI network was visualized by using Cytoscape software (version 3.7.2). Core genes were identified using the cytoHubba plug-in based on 10 algorithms (33).

### Receiver operating characteristic curve analysis

ROC analysis was performed to further assess the predictive accuracy of core genes. The pROC package was applied to draw the ROC curves of core genes based on the gene expression profile.

### Enrichment analysis

Gene set enrichment analysis (GSEA) is often used to analyze and interpret pathway-level changes between normal and disease groups (34). The “clusterProfiler”, “enrichplot”, “pathwork”, and “DOSE” packages of R were applied to implement GSEA. The gene set of “h.all.v7.3.symbols” from the Molecular Signature Database was used as a reference gene set. *P* < 0.05 was considered a significant enrichment. Gene Set Variation analysis (GSVA) was also conducted by using the tidyverse R package and “C2.cp.all.v7.0.symbols” was used as the reference gene set.

### Estimation of immune cell infiltrations in DN

xCell is a robust algorithm that analyzes the infiltration levels of 64 immune and stroma cell types, including extracellular matrix cells, epithelial cells, hematopoietic progenitors, innate and adaptive immune cells (27). The xCell algorithm was used to analyze the immune cell infiltrations between two subgroups. The correlation analysis between core genes and immune cell types was performed using the ggplot2 package of R.

## Results

### Screening for DEGs

**Figure 2:**
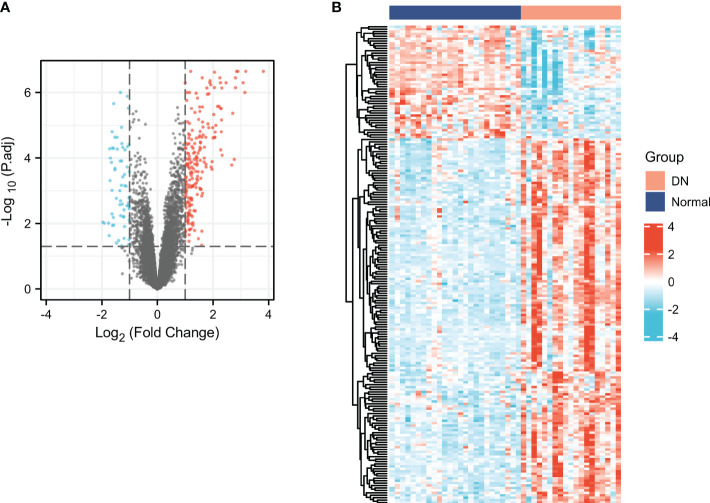
Screening for DEGs in DN. Volcano **(A)** and heatmap **(B)** plots of the DEGs. For the volcano, the blue dots indicated the down-regulated genes and the red dots indicated the up-regulated genes. For the heatmap, the red group is the DN group, while the dark blue group is the normal group. The down-regulated genes are presented in blue, and up-regulated genes are presented in red.

As presented in **Figure 2A**, we identified a total of 266 DEGs between normal and DN groups, of which 202 genes were up-regulated and 64 genes were down-regulated. The cluster heatmap of the DEGs was presented in **Figure 2B**.

### Characterization of the immune cell infiltrations in DN and identification of ISRGs

**Figure 3:**
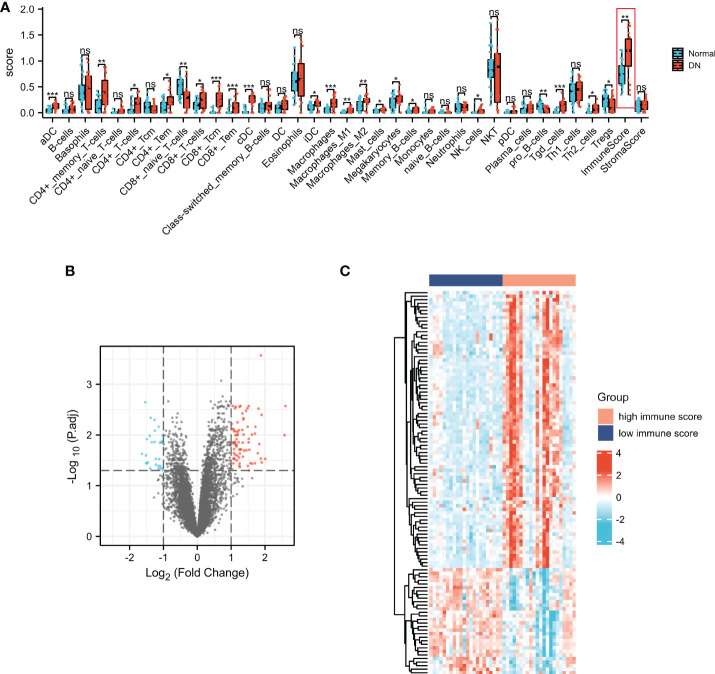
Characterization of the immune cell infiltrations in DN and identification of ISRGs. **(A)** The comparisons of the immune cell infiltration level between DN and normal groups. Volcano **(B)** and heatmap **(C)** plots of the ISRGs. For the volcano, the blue dots indicated the down-regulated genes and the red dots indicated the up-regulated genes. For the heatmap, the red group is the high immune score group, while the dark blue group is the low immune score group. The down-regulated genes are presented in blue, and up-regulated genes are presented in red.

xCell was applied to assess the differences in immune cell infiltrates between the DN and normal groups. As shown in **Figure 3A**, aDC, CD4+ memory T cells, CD4+ T cells, CD4+ Tem, CD8+ T cells, CD8+ Tcm, CD8+ Tem, cDC, iDC, macrophages, macrophages M1, macrophages M2, mast cells, memory B cells, NK cells, Tgd cells, Th2 cells, and immune score were significantly increased in the DN group, whereas CD8+naïve T cells, pro B cells, and Tregs were significantly decreased in the DN group. The results indicated that immune cell patterns were significantly different between the normal and DN groups. Thus, two immune score-related subgroups were divided based on the median values of the immune score. As presented in **Figure 3B**, a total of 108 ISRGs were obtained between high immune score and low immune score subgroups. Among these genes, 78 genes were up-regulated, and 30 genes were down-regulated. The heatmap of the IRGs were presented in **Figure 3C**.

### Functional enrichment analysis of ISRDEGs

**Figure 4:**
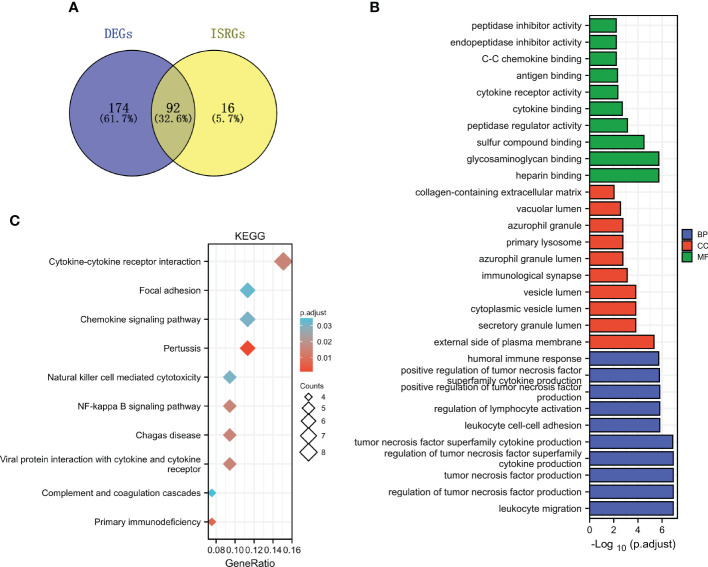
Functional enrichment analysis of ISRDEGs. **(A)** The Venn diagram shows the interaction between DEGs and ISRGs. GO **(B)** and KEGG **(C)** enrichment analyses for the 92 ISRDEGs.

The common genes (ISRDEGs) between DEGs and ISRGs were identified (**Figure 4A**). Based on the Venn result, a total of 92 ISRDEGs were used to perform the functional enrichment analysis. As shown in **Figure 4B** and **Table S1**. The results indicated that ISRDEGs were mainly enriched in the GO-biological process (BP) of leukocyte migration, regulation of lymphocyte activation, leukocyte cell-cell adhesion, tumor necrosis factor superfamily cytokine production, regulation of tumor necrosis factor superfamily cytokine production, etc. ISRDEGs were mainly enriched in GO-cell component (CC) of secretory granule lumen, immunological synapse, primary lysosome, azurophil granule, etc. ISRDEGs were mainly enriched in GO-molecular function (MF) of heparin-binding, glycosaminoglycan binding, peptidase regulator activity, cytokine binding, C-C chemokine binding, etc. Besides, the results of KEGG enrichment analysis showed that the 92 ISRDEGs were significantly enriched in cytokine-cytokine receptor interaction, focal adhesion, chemokine signaling pathway, pertussis, natural killer cell medicated-cytotoxicity, NF-kappa B signaling pathway, Chagas disease, viral protein interaction with cytokine and cytokine receptor, and primary immunodeficiency (**Figure 4C** and **Table S1**).

### Unsupervised cluster analysis identifies immune-associated gene subtypes

**Figure 5:**
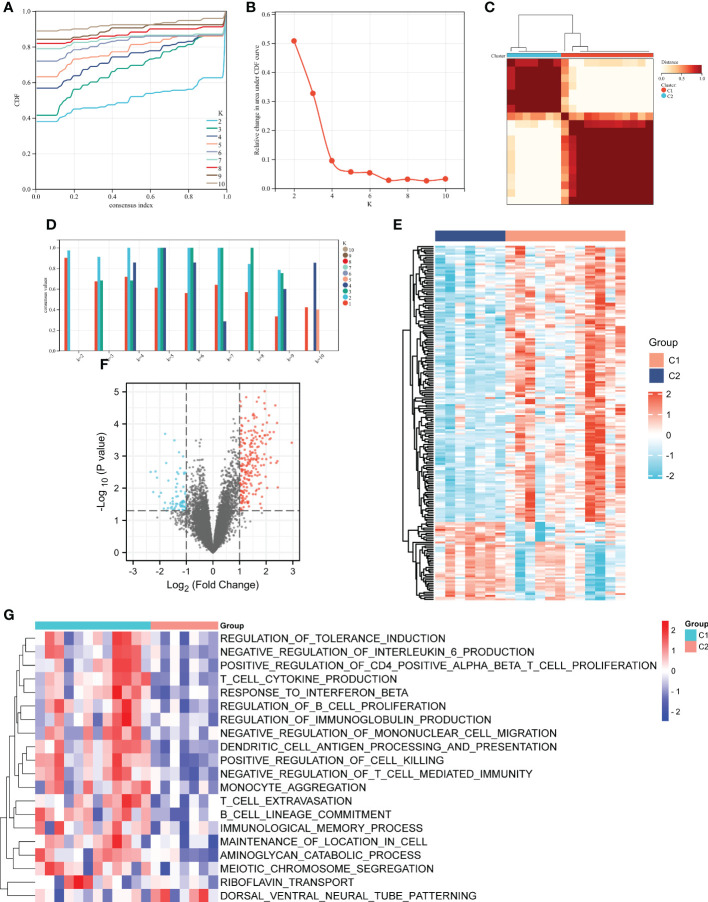
Unsupervised clustering analysis of immune-associated genes. **(A)** Consensus CDF when k = 2-10. **(B)** The cophenetic correlation coefficient was calculated for k = 2-10. **(C)** Consensus values when k = 2-10. **(D)** Heatmap of the matrix of co-occurrence proportions for DN samples. Heatmap **(E)** and volcano **(F)** and plots of the differential genes between C1 and C2 subgroups. **(G)** Heatmap of the potential pathways between C1 and C2 subgroups by GSVA. For the volcano, the blue dots indicated the down-regulated genes and the red dots indicated the up-regulated genes. For the heatmap, the down-regulated genes or pathways are presented in blue, and up-regulated genes or pathways are presented in red.

The unsupervised cluster analysis was applied to classify the DN patients with different immune phenotypes based on the expression of 92 ISRDEGs. We used the cophenetic correlation coefficients to determine the optimal k number, and our results showed that k = 2 was the optimal subtype number (**Figures 5A–D**). Two subtypes of DN samples were identified: C1 with 12 samples, and C2 with 7 samples. As presented in **Figures 5E, F**, a total of 286 differential genes were obtained between C1 and C2 subgroups. Among these genes, 218 genes were up-regulated, and 68 genes were down-regulated. As shown in **Figure 5G**, the positive regulation of CD4 positive alpha beta T cell proliferation, T cell cytokine production, response to interferon beta, regulation of B cell proliferation, regulation of immunoglobulin production, T cell extravasation, B cell lineage commitment, and immunological memory process were activated in C1 subgroup, indicating that the C1 subgroup exhibited higher immune activation than the C2 subgroup.

### Identification of key modules by WGCNA

**Figure 6:**
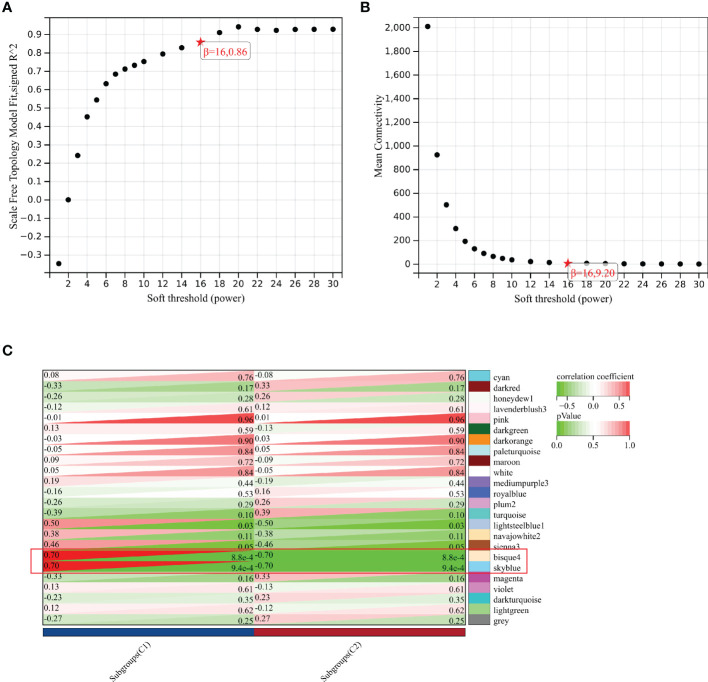
Identification of key module by WGCNA. **(A)** Scale-free fitting index analysis for the different soft thresholds. **(B)** Mean connectively for the different soft thresholds. **(C)** Heatmap presenting the module-trait correlations.

As shown in **Figures 6A, B**, the soft-threshold power of β = 16 was selected, and the corresponding Pearson’s correlation coefficient was calculated to construct a scale-free network. As shown in **Figure 6C**, 24 modules were identified, and bisque4 and skyblue modules exhibited the highest correlation with subgroups. Therefore, we selected the two modules for further analysis.

### Identification and functional enrichment analysis of IRGs in key modules

**Figure 7:**
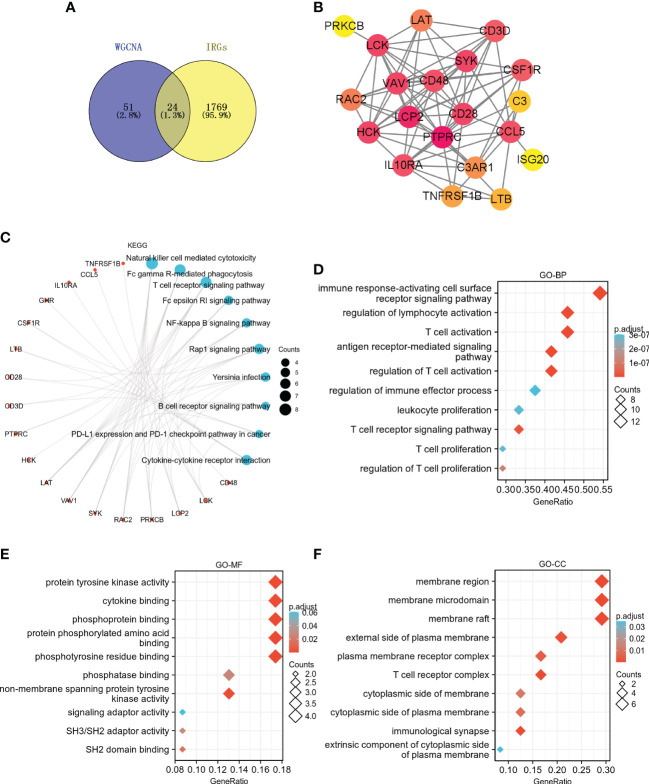
Identification and functional enrichment analysis of IRGs in key modules. **(A)** The Venn diagram shows the interaction between the key modules and IRGs. **(B)** PPI network of 24 IRGs. KEGG **(C)**, GO-BP **(D)**, GO-MF **(E)**, and GO-CC **(F)** enrichment analyses for the 24 IRGs.

75 hub genes were identified in the two key modules (bisque4 and skyblue) based on MM > 0.8 and GS > 0.6. Among these hub genes, 24 IRGs were identified (**Figures 7A, B** **)**. KEGG pathway analysis showed that these 24 IRGs were mainly enriched in natural killer cell mediated cytotoxicity, T cell receptor signaling pathway, NF-kappa B signaling pathway, Rap1 signaling pathway, B cell receptor signaling pathway, PD-L1 expression and PD-1 checkpoint pathway in cancer, cytokine-cytokine receptor interaction, etc (**Figure 7C**). These 24 IRGs in BP were mainly involved in immune response-activating cell surface receptor signaling pathway, regulation of lymphocyte activation, T cell activation, leukocyte proliferation, T cell receptor signaling pathway, T cell proliferation, etc (**Figure 7D**). As for MF, 24 IRGs were mainly enriched in protein tyrosine kinase activity, cytokine binding, phosphoprotein binding, etc (**Figure 7E**). The CC analysis showed that 24 IRGs were enriched in the membrane region, membrane microdomain, plasma membrane receptor complex, T cell receptor complex, etc (**Figure 7F**).

### Identification and verification of core genes

**Figure 8:**
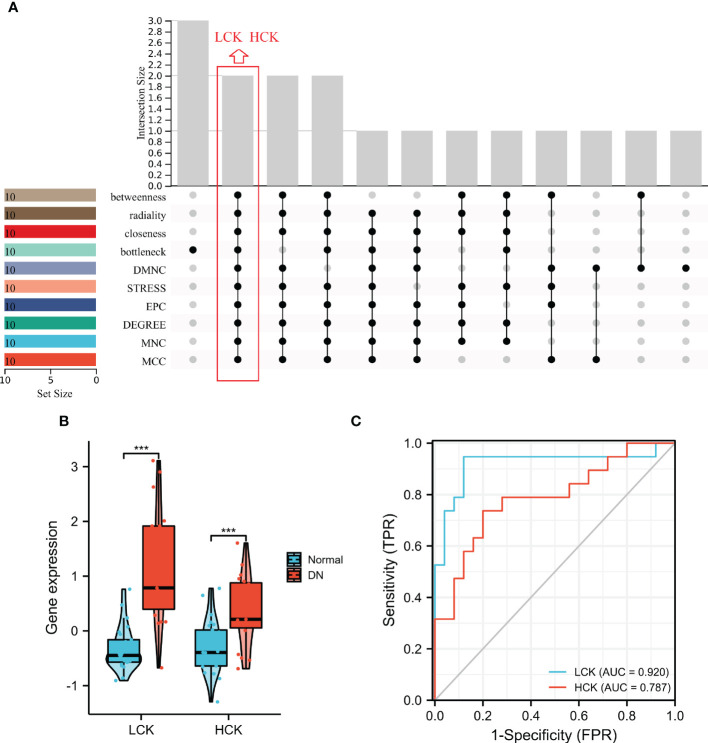
Identification of core genes. **(A)** Two core genes were identified by ten algorithms. **(B)** LCK and HCK gene expression in the training dataset. ***p < 0.001 was analyzed by the Willcoxon rank sum test. **(C)** ROC curves of LCK and HCK genes in the training dataset.

Two core genes, namely, lymphocyte-specific protein tyrosine kinase (LCK) and hematopoietic cell kinase (HCK) were identified based on the overlapped parameters of the top 10 IRGs in 10 algorithms (**Figure 8A**). As shown in **Figure 8B**, the expression levels of LCK and HCK genes in the normal group were significantly lower than that of the DN group (p < 0.001). Besides, the diagnostic AUC values of LCK and HCK genes were 0.92 and 0.787, respectively (**Figure 8C**).

**Figure 9:**
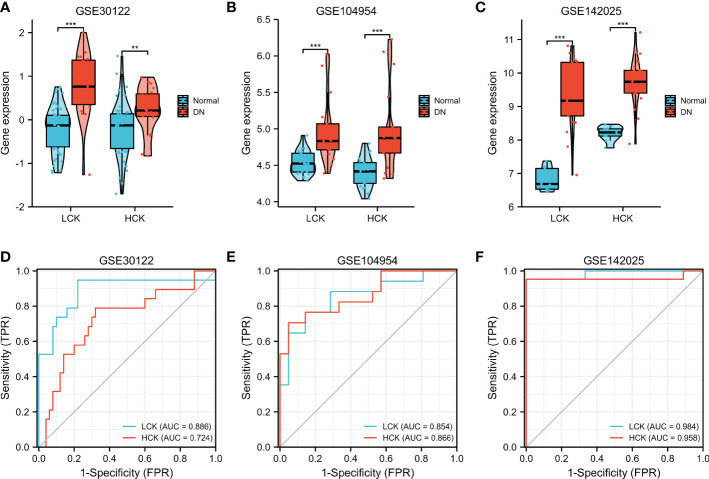
Validation of core genes in DN. Verification of LCK and HCK gene expression in the GSE30122 **(A)**, GSE104954 **(B)**, and GSE142025 datasets **(C)**. **p < 0.01 and ***p < 0.001 were analyzed by the Willcoxon rank sum test. ROC curves of LCK and HCK genes in the GSE30122 **(D)**, GSE104954 **(E)**, and GSE142025 datasets **(F)**.

Furthermore, three independent microarray datasets (GSE30122, GSE142025, and GSE104954) were used for cross-validation. As shown in **Figures 9A–C**, the expression levels of LCK and HCK genes in the normal group were significantly lower than that of the DN group (p < 0.01). Besides, the diagnostic value of core genes was verified by ROC analysis. For the GSE30122 cohort, the diagnostic AUC values of LCK and HCK genes were 0.886 and 0.724, respectively (**Figure 9D**). For the GSE104954 cohort, the diagnostic AUC values of LCK and HCK genes were 0.854 and 0.866, respectively (**Figure 9E**). For the GSE142025 cohort, diagnostic AUC values of LCK and HCK genes were 0.984 and 0.958, respectively (**Figure 9F**).

### Single gene GSEA

**Figure 10:**
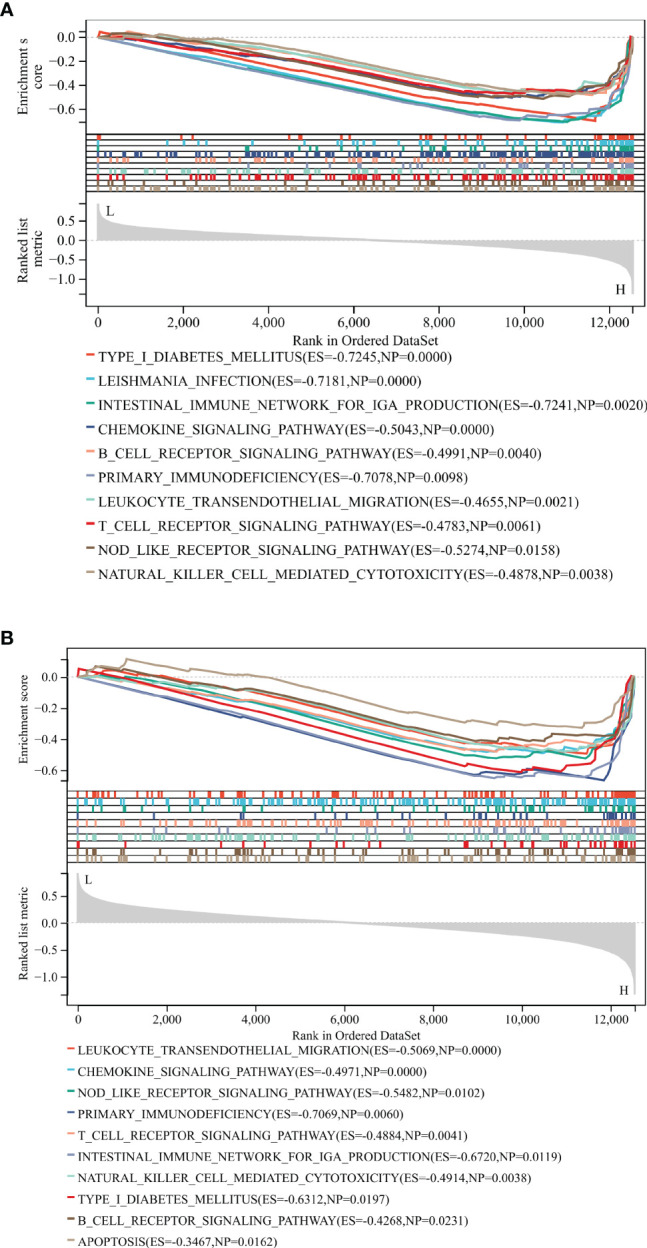
GSEA identified potential signaling pathways associated with the core genes. The major signaling pathways are mainly enriched in high expressions of HCK **(A)** and LCK **(B)**.

We analyzed potential signaling pathways associated with core genes *via* GSEA. As shown in **Figure 10A**, type 1 diabetes mellitus (NES = -0.7254, p < 0.01), leishmania infection (ES = -0.7881, p < 0.01), intestinal immune network for IGA production (NES = -0.724, p = 0.002), chemokine signaling pathway (NES = -0.504, p < 0.001), B cell receptor signaling pathway (NES = -0.49, p = 0.004), primary immunodeficiency (NES = -0.7078, p = 0.009), leukocyte transendothelial migration (NES = -0.4655, p = 0.002), T cell receptor signaling pathway (NES = -0.4783, p = 0.006), NOD-like receptor signaling pathway (NES = -0.5274, p = 0.01), and natural killer cell-mediated cytotoxicity (NES = -0.4878, p = 0.003) were mainly enriched in the HCK high-expressed phenotype.

As shown in **Figure 10B**, leukocyte transendothelial migration (NES = -0.5069, p < 0.001), chemokine signaling pathway (NES = -0.4971, p < 0.001), NOD-like receptor signaling pathway (NES = -0.5482, p = 0.01), primary immunodeficiency (NES = -0.7069, p = 0.006), T cell receptor signaling pathway (NES = -0.4884, p = 0.004), intestinal immune network for IGA production (NES = -0.724, p = 0.002), natural killer cell-mediated cytotoxicity (NES = -0.4914, p = 0.0038), Type 1 diabetes mellitus (NES = -0.6312, p = 0.019), B cell receptor signaling pathway (NES = -0.4268, p = 0.023), and apoptosis (ES = -0.3467, p = 0.016) were mainly enriched in the LCK high-expressed phenotype. These results revealed that the two core genes were all linked to the immune responses.

### Immune cell infiltration analysis

**Figure 11:**
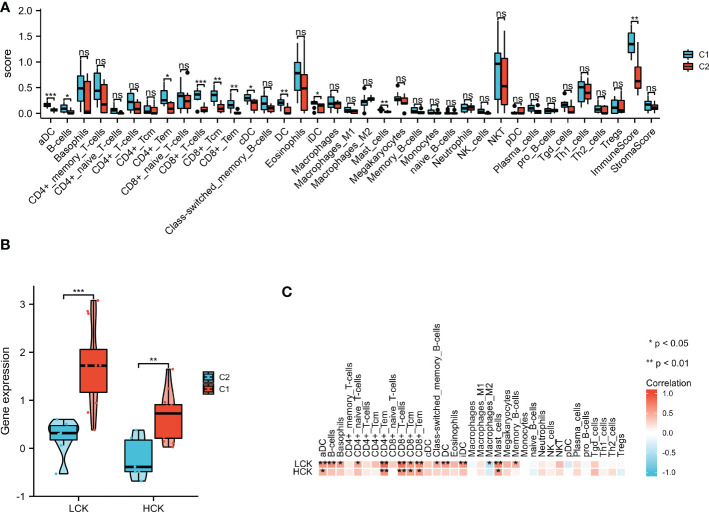
xCell algorithm was used to perform immune cell infiltration analysis. **(A)** The landscape of the 35 types of immune cells between C1 and C2 subgroups. **(B)** LCK and HCK gene expression between C1 and C2 subgroups. **(C)** Correlation analysis between the core genes and 35 immune cell infiltration levels. *p < 0.05; **p < 0.01; ***p < 0.001; ns, no statistical difference.

xCell was applied to assess the differences in immune cell infiltrates between the C1 and C2 subgroups. As shown in **Figure 11A**, aDC, B cells, CD4+ Tem, CD8+ T cells, CD8+ Tcm, CD8+ Tem, cDC, dendritic cells (DC), iDC, mast cells, and immune score were significantly increased in the C1 subgroup. As shown in **Figure 11B**, the expression levels of LCK and HCK genes in the C1 subgroup were significantly higher than that of the C2 subgroup (p < 0.01). Besides, correlation analysis showed that LCK and HCK were positively correlated with aDC, CD4+ Tem, CD8+ T cells, CD8+ Tem, CD8+ Tcm, and mast cells (**Figure 11C**).

## Discussion

DN is one of the common microvascular complications of type 2 diabetes mellitus and occurs in up to 20-50% of patients with diabetes (35). DN imposes a huge burden on national health systems due to its high expenditure and high incidence. Therefore, there is an urgent need to develop promising clinical biomarkers of early DN that can effectively slow the progression of DN. DN represents a chronic inflammatory disease that leads to renal function disorder, albuminuria, and podocyte injury (11). Recent studies have revealed that inflammation and immune pathways play important roles in DN progression, and the identification of novel immune-related signatures may link to the development of novel therapeutic strategies (8, 19, 36). Thus, it is important to develop novel immune-associated signatures for the diagnosis and treatment of DN.

In our study, we identified 92 ISRDEGs in the expression profiles of 25 normal samples and 19 DN samples using a series of bioinformatics analyses. The subsequent KEGG and BP functional enrichment analyses indicated that these ISRDEGs were enriched in leukocyte migration, regulation of lymphocyte activation, humoral immune response, leukocyte cell-cell adhesion, tumor necrosis factor superfamily cytokine production, cytokine-cytokine receptor interaction, chemokine signaling pathway, natural killer cell mediated cytotoxicity, NF-kappa B signaling pathway, and primary immunodeficiency. Consistent with our findings, activated leukocytes play an important role in the pathogenesis of DN (37). Cytokine and cytokine receptors have been reported to play a key role in macrophage/monocyte recruitment in animal models of DN, as well as associated with the progression of interstitial inflammation of DN (38, 39).

Based on the result of WGCNA, two IRGs (LCK and HCK) were identified as core biomarkers associated with immune response in DN. All of them could predict the progression of DN in multiple GEO datasets. LCK is a vital activator of T cells, which plays an important role in several cellular processes such as cell differentiation, cell proliferation, cell adhesion, and cell cycle (40, 41). LCK is related to the CD8 and CD4 co-receptors and contributes to signaling *via* T cell receptor complex (42). Since LCK plays a vital role in T cell signaling and cytokine production, alteration in activity or expression of LCK may be involved in various disease progressions, including atherosclerosis, ulcerative colitis, psoriasis, rheumatoid arthritis, diabetes, asthma, and cancer (41). LCK is involved in the progress of leptin-induced renal inflammation during aging (43). A recent study revealed that the G allele of SNP rs10914542 of LCK inhibits the TCR/CD3-mediated T cell activation and increases the risk of type 1 diabetes in children (44). It has been reported that βig-h3 inhibits T cell activation in type 1 diabetes *via* suppression of LCK (45). HCK transmits various extracellular signals and involves in cell migration, cell differentiation, and cell proliferation (46). Activation of the NLRP3 inflammasome may involve the development of DN (47). HCK is essential for the activation of NLRP3 inflammasome *in vivo* (48). The previous study revealed that HCK is an important Src kinase family member that participated in the progression of renal fibrosis (49). HCK plays a vital role in the macrophage activation and the secretion of TNF-α, which leads to the progression of diabetes (50). However, there were few reports on the role of LCK and HCK in DN progression. In the present study, for the first time, our findings showed that LCK and HCK were significantly up-regulated in DN and could act as an effectively diagnostic biomarker for DN patients.

Importantly, LCK and HCK were also found to be positively correlated with immune cell infiltrations in DN progression. Recent studies revealed that DN is a chronic inflammatory disease, and immune cells associated with adaptive and innate immune responses, including mast cells, neutrophils, T cells, B cells, DC, and macrophage, might be implicated in DN development (51). Since the immune-associated pathways (positive regulation of CD4 positive alpha beta T cell proliferation, T cell cytokine production, regulation of B cell proliferation, regulation of immunoglobulin production, T cell extravasation, B cell lineage commitment, and immunological memory process) were activated in the C1 subgroup, the relationship between DN subgroups and immune cells was further investigated. We found that the aDC, B cells, CD4+ Tem, CD8+ T cells, CD8+ Tcm, CD8+ Tem, cDC, DC, iDC, mast cells, and immune score were significantly expressed in the C1 subgroup compared with the C2 subgroup. The previous study has reported that aberrant recruitment and activation of T cells in the interstitium are the potential pathological mechanisms of DN (52). B cells also contributed to the progression of diabetic kidney disease (53). Mast cells could promote renal fibrosis and inflammation, and thus implicated in the pathogenesis of DN (54, 55). A recent study reported that DC plays an important role in the pathogenesis of DN (56). Therefore, we speculated the C1 subgroup is more likely to develop advanced DN than the C2 subgroup.

However, there are several limitations in our study. First, larger clinical sample sizes are needed to verify the expression of key genes. Second, DN-related animal models should be established to verify the role of immune-related genes on disease progression.

## Conclusion

In conclusion, we identified two core genes (LCK and HCK) as diagnostic biomarkers for the diagnosis and immunotherapy of DN patients. Furthermore, we proposed a novel molecular classification containing non-immune and immune subgroups in DN patients. Collectively, our findings could help to design more precise tailored immunotherapy for ND patients.

## Data availability statement

The original contributions presented in the study are included in the article/**Supplementary Material**. Further inquiries can be directed to the corresponding authors.

## Author contributions

KL and LW wrote the manuscript and designed the study. YF, GL and MC analyzed the data. XZ reviewed and edited the manuscript. All authors contributed to the article and approved the submitted version.
